# Development of the nervous system in *Phoronopsis harmeri* (Lophotrochozoa, Phoronida) reveals both deuterostome- and trochozoan-like features

**DOI:** 10.1186/1471-2148-12-121

**Published:** 2012-07-24

**Authors:** Elena Temereva, Andreas Wanninger

**Affiliations:** 1 Department of Invertebrate Zoology, Biological faculty, Moscow State University, Moscow, 119991, Russia; 2 Dept. of Integrative Zoology, University of Vienna, Althanstr 14, 1090, Vienna, Austria

**Keywords:** Neurogenesis, Evolution, Phylogeny, Ventral nerve cord, Last common lophotrochozoan ancestor

## Abstract

**Background:**

Inferences concerning the evolution of invertebrate nervous systems are often hampered by the lack of a solid data base for little known but phylogenetically crucial taxa. In order to contribute to the discussion concerning the ancestral neural pattern of the Lophotrochozoa (a major clade that includes a number of phyla that exhibit a ciliated larva in their life cycle), we investigated neurogenesis in *Phoronopsis harmeri*, a member of the poorly studied Phoronida, by using antibody staining against serotonin and FMRFamide in combination with confocal microscopy and 3D reconstruction software.

**Results:**

The larva of *Phoronopsis harmeri* exhibits a highly complex nervous system, including an apical organ that consists of four different neural cell types, such as numerous serotonin-like immunoreactive flask-shaped cells. In addition, serotonin- and FMRFamide-like immunoreactive bi- or multipolar perikarya that give rise to a tentacular neurite bundle which innervates the postoral ciliated band are found. The preoral ciliated band is innervated by marginal serotonin-like as well as FMRFamide-like immunoreactive neurite bundles. The telotroch is innervated by two neurite bundles. The oral field is the most densely innervated area and contains ventral and ventro-lateral neurite bundles as well as several groups of perikarya. The digestive system is innervated by both serotonin- and FMRFamide-like immunoreactive neurites and perikarya. Importantly, older larvae of P. harmeri show a paired ventral neurite bundle with serial commissures and perikarya.

**Conclusions:**

Serotonin-like flask-shaped cells such as the ones described herein for *Phoronopsis harmeri* are found in the majority of lophotrochozoan larvae and therefore most likely belong to the ground pattern of the last common lophotrochozoan ancestor. The finding of a transitory paired ventral neurite bundle with serially repeated commissures that disappears during metamorphosis suggests that such a structure was part of the “ur-phoronid” nervous system, but was lost in the adult stage, probably due to its acquired sessile benthic lifestyle.

## Background

Although the exact phylogenetic position of Phoronida is still a matter of ongoing debate (and in some recent studies even their monophyly is questioned, e.g., [1,2]), all recent molecular analyses agree in their inclusion within the protostome superclade Lophotrochozoa [3-5]. Mainly based on a proposed homologous feeding organ, the lophophore, Phoronida has been traditionally aligned with Ectoprocta and Brachiopoda to form the monophyletic Lophophorata [6,7]. This view is supported by a shared radial type of cleavage [8] (although some unpublished observations claim that spiral cleavage may be present in some phoronids), but otherwise there is little morphological support for such an assemblage, mainly due to the high disparity of the adult bodyplans of these phyla. The same is true for the proposed lophophorate-spiralian alignment, where autapomorphies are hard to come by, one of them may be the occurrence of a larval apical organ with serotonin-like immunoreactive flask-shape cells [9]. On the other hand, lophophorates share some embryological features with the seemingly distantly related deuterostomes, such as the above-mentioned radial cleavage [8,10], the (sometimes questioned) three coelomic compartments [11], and an upstream ciliary filter feeding system [12,13].

The nervous system is often considered highly conserved between major animal taxa and is therefore often used for morphology-based phylogenetic inferences [14]. While an impressive amount of data on the development of the nervous system has recently become available for numerous lophotrochozoan subtaxa, phoronid neurogenesis is still comparatively little known, with most studies dealing with the description of the neuroanatomy of more or less mature larvae rather than with the development of the nervous system as such [15-21]. There are exceptions to the rule, however, but the few studies on phoronid neural development are restricted to one species belonging to the genus *Phoronis* and are not detailed enough to allow for answers concerning crucial issues such as the exact cellular composition and arrangement of the apical organ, thus rendering comparisons with other lophophorates, or even spiralians, difficult. Accordingly, a reconstruction of the neural anatomy of the larva of the last common lophophorate (and therefore lophotrochozoan) ancestor remains highly speculative. The unexpected recent finding of a paired ventral neurite bundle in the actinotroch larva of *Phoronopsis harmeri*[22] similar to most spiralians may provide evidence for such a neural system in the phoronid as well as lophotrochozoan groundplan. Such a scenario would imply, however, secondary loss of this paired ventral nervous system in adult phoronids (probably as a result of a sessile lifestyle), since they do not exhibit a comparable neural structure. In order to broaden the database on phoronid neurogenesis and larval neuroanatomy and to contribute to the discussion concerning the groundpattern of the phoronid and lophotrochozoan neural bauplan, we investigated the development of the serotonin-like and FMFRamide-like immunoreactive nervous system during larval development of *Phoronopsis harmeri*.

## Results

### Description of major developmental stages

**Figure 1:**
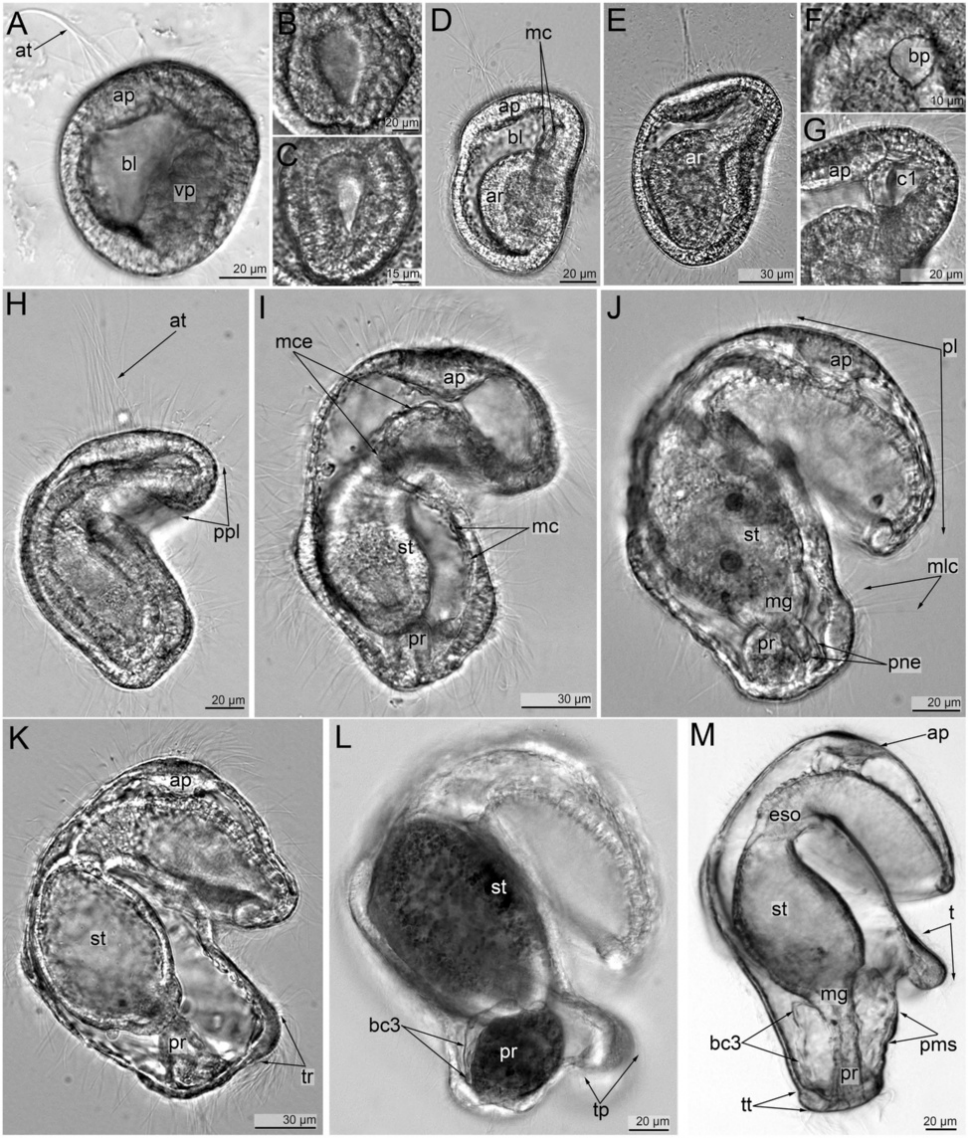
**Embryonic and larval development of *Phoronopsis harmeri* (photographs of live animals).** Whole embryos and larvae are in lateral right view with anterior up (**A**) The early gastrula has a spacious blastocoel (bl), a flat vegetal pole (vp), an apical plate (ap), and an apical tuft (at). (**B**) Rounded blastopore of the early gastrula. (**C**) Flask-shaped blastopore of a mid-gastrula stage. (**D**) Ventral side of a mid- gastrula with rounded archenteron (ar) and mesodermal cells (mc) in the blastocoels above the archenteron. (**E**) The late gastrula I has a large apical plate, an elongate archenteron (ar), and a small coelomic cavity in the anterior body part. (**F**) A tear-shaped blastopore (bp) of the late gastrula I. (**G**) The anterior body part of the late gastrula I with coelomic cavity (c1) and apical plate (ap). (**H**) The late gastrula II shows the precursor of the preoral lobe (ppl), which resembles an epidermal fold above the mouth. (**I**) The preactinotrocha has an apical plate (ap), stomach (st), proctodeum (pr), two tiers of the mesodermal cells (mc), and well developed muscles of the esophagus (mce). (**J**) The young actinotrocha is the first feeding stage with a well-developed preoral lobe (pl), complete intestine with stomach (st) and proctodaeum (pr), motionless cilia (mlc) along the tentacular ridge, and two protonephridial channels (pne). (**K**) A 6-day-old actinotrocha has a well-developed tentacular ridge (tr) and a short trunk. (**L**) A 13-day-old actinotrocha has precursors of the tentacles (pt) along the tentacular ridge, a longer trunk, and a small trunk coelom (borders are indicated as bc3). (**M**) The actinotroch larva with three pairs of tentacles has six short tentacles (t), complete intestine with esophagus (eso), stomach (st), midgut (mg), and proctodaeum (pr), a long trunk with trunk coelom, a terminal telotroch (tt), and the precursor of the metasomal sack (pms).

The development of *Phoronopsis harmeri* was previously described in detail [10]. We briefly describe here the gross morphological characters of the crucial stages of larval development. The apical plate, which later includes the apical organ with an apical ciliary tuft, appears in the early gastrula (approximately 18 h post spawning (hps)), which has a flattened vegetal pole and a round blastopore (Figure 1A, B). The first immunoreactive cells differentiate only in the mid-gastrula stages (approximately 32 hps).

Mid-gastrula (approximately 30–37 hps): this stage is 140 μm long and 100 μm wide. The archenteron is rounded (Figure 1D). The blastopore is flask-shaped with swollen anterior and narrow posterior portions (Figure 1C). At this stage, the anterior mesodermal precursor resembles a large mass of cells in front of and on both sides of the archenteron (Figure 1D).

Late gastrula I (approximately, 40–47 hps): this stage has an elongated archenteron with a cone-shaped posterior portion (Figure 1E). It represents the anlage of the future midgut. The blastopore closes and becomes tear-shaped with a narrow posterior portion (Figure 1F). The volume of the blastocoel decreases in the posterior part of the embryo. Cells of the anterior coelomic precursor often form processes that pass to the apical plate, and the preoral coelom forms in the anterior part of the embryo (Figure 1G).

Late gastrula II (approximately 48–52 hps): the body shape of this stage differs from that of the previous stage in that the mouth becomes deeper and the precursor of the preoral lobe forms (Figure 1H). The latter is an epidermal fold that is formed by two epidermal layers above the mouth. On the lateral and ventral sides of the posterior portion of the body, the postoral ciliated band, which is a belt of thick epithelium, appears. At this stage, the midgut contacts the surface of the epidermis of the posterior body part.

Preactinotrocha (approximately, 55–63 hps.): At this stage, the proctodaeum appears but the larva is still lecithotrophic (Figure 1I). The larva has a well-developed esophageal muscle, the preoral coelom, and the postoral ciliated band, which does not yet contain motionless latero-frontal cilia.

Young actinotrocha (approximately, 63–70 hps.): This stage has a well-developed preoral lobe (Figure 1J). The preoral lobe is the large anterior portion of the larva and has a large blastocoel, which is crossed by processes of mesodermal cells. The latero-frontal cilia on the tentacular ridge appear and the larva starts to feed. On both sides of the anus, small epidermal invaginations arise, which form the protonephridial tubes.

5- to 6-day-old actinotrocha: The larva has large blastocoels in the hyposphere, a short trunk under the tentacular ridge, and well-developed two protonephridia with several terminal cells (Figure 1K).

13-day-old actinotrocha: Specimens feed, increase in size, and retain nutrients in the cells of the stomach and hindgut (Figure 1L). There are three pairs of protrusions along the tentacular ridge, the primordial tentacles.

Actinotrocha with three pairs of tentacles (approximately 24 days after spawning): The larva has a well-developed trunk with a terminal telotroch around the anus (Figure 1M). The ventral pair of tentacles is the longest, and the dorso-lateral pair is the shortest. The trunk is occupied by a large trunk coelom. On the ventral body side under the tentacles, an area of thick epidermis appears which forms the primordium of the metasomal sac.

### Neurogenesis

Although the apical tuft and the apical plate form in the early gastrula, perikarya do not differentiate and connect to the sensory cells of the apical plate until the mesodermal muscle cells appear, which is in the mid-gastrula stage.

#### Serotonin-like immunoreactive nervous system

**Figure 2:**
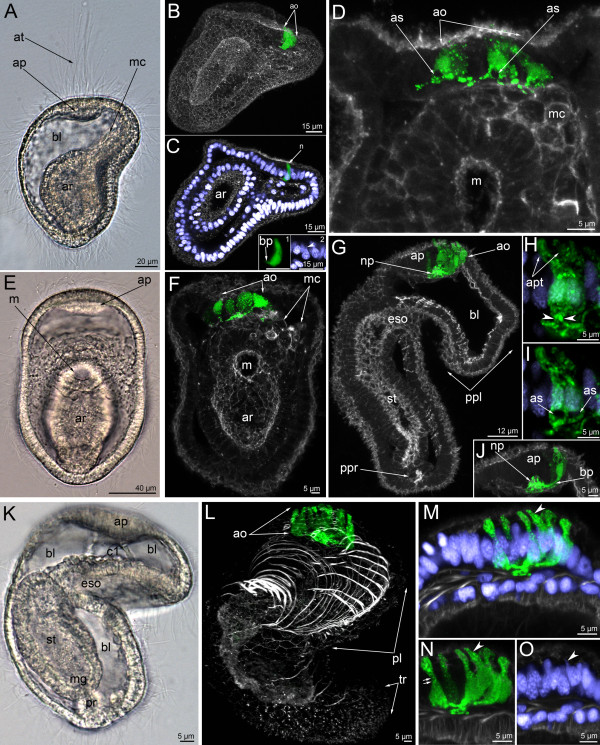
**The organization of the serotonin-like immunoreactive nervous system in the mid-gastrula (A-D), late gastrula I (E-F), late gastrula II (G-J), and preactinotrocha (K-O).** Photographs of live animals (A, E, K) and Z-projections of embryos after mono-, double, and triple staining for 5-HT (serotonin) (green), phalloidin (grey), and Hoechst (violet). Apical is to the top on all micrographs except for B and C, where it is to the upper right. (**A**) Mid-gastrula gross anatomy showing the apical plate (ap), the archenteron (ar), the apical tuft (at), the blastocoel (bl), and the mesodermal cells (mc); lateral view, ventral side is to the right. (**B**) Overview of a mid-gastrula stage with apical organ (ao). (**C**) The same embryo with one perikaryon (n). Image created from selected optical sections from the mid region of the specimen. (C-insert 1) Detail of a perikaryon with short basal process (bp). (C-insert 2) Detail of the nucleus of the perikaryon, which has an upper thin protrusion (arrowhead). (**D**) Anterior portion of a mid-gastrula with apical organ, which consists of four perikarya. Some perikarya have a special area (as) devoid of signal under the wide part of the cell containing the nucleus. Ventral view showing mesodermal cells (mc) and mouth (m). (**E**) Ventral view of live late gastrula I showing apical plate, mouth, and archenteron. (**F**) Serotonin-like immunoreactive nervous system in late gastrula I: the apical organ is formed by 6–7 perikarya. Ventral view showing mesodermal cells, mouth, and archenteron. Image created from selected optical sections from the mid region of the specimen. (**G**) Optical sagittal section through a late gastrula II, which has a large apical organ, the precursor of the preoral lobe (ppl) with spacious blastocoels (bl) and apical plate, esophagus (eso), stomach (st), and the precursor of the proctodaeum (ppr). (H-J) Detail of perikarya of a late gastrula II. (**H**) Two perikarya with two separated apical parts (apt) and one basal process (arrowheads). (**I**) Two perikarya with special areas (as), which are located under the nuclei and lack staining. (**J**) Small neuropil (np) and perikaryon with long basal neurite (bp). (**K**) Lateral view (ventral side on the right) of a live preactinotrocha with complete digestive tract, which consists of esophagus, stomach, midgut (mg), proctodaeum (pr), and small preoral coelom (c1) under the apical plate and blastocoel (bl) in the preoral lobe. (**L**) Overview of a preactinotrocha, dorso-lateral view showing apical organ, preoral lobe, and tentacular ridge. (M-O) Details of the organization of parikarya of the apical organ. Arrowhead indicates an individual perikaryon, which has a nucleus with apical protrusion. (**M**) General view of perikarya. (**N**) Body shape of perikarya with constricted area (double arrow) between the apical and basal parts. (**O**) Nuclei of perikarya; arrowhead indicates apical protrusion of one nucleus.

During the mid-gastrula stage, the first perikarya differentiate in the epithelium of the apical plate (Figure 2A-C). Four serotonin-like immunoreactive flask-shaped perikarya usually differentiate simultaneously (Figure 2D, 3A, B). They are located at the anterior edge of the apical plate and have a thickened basal part and a narrow apical part (Figure 2C-insert 1, 3B). The apical part contacts the surface and bears the cilium. The basal part of each cell forms short processes that project towards the central part of the apical plate (Figure 2C, C-insert 1). Most somatic cells of the embryo have a tear-shaped nucleus with a wide basal part and a thin apical part (Figure 2C). This form of nucleus is also typical for serotonin-like immunoreactive perikarya (Figure 2C-insert 2).

**Figure 3:**
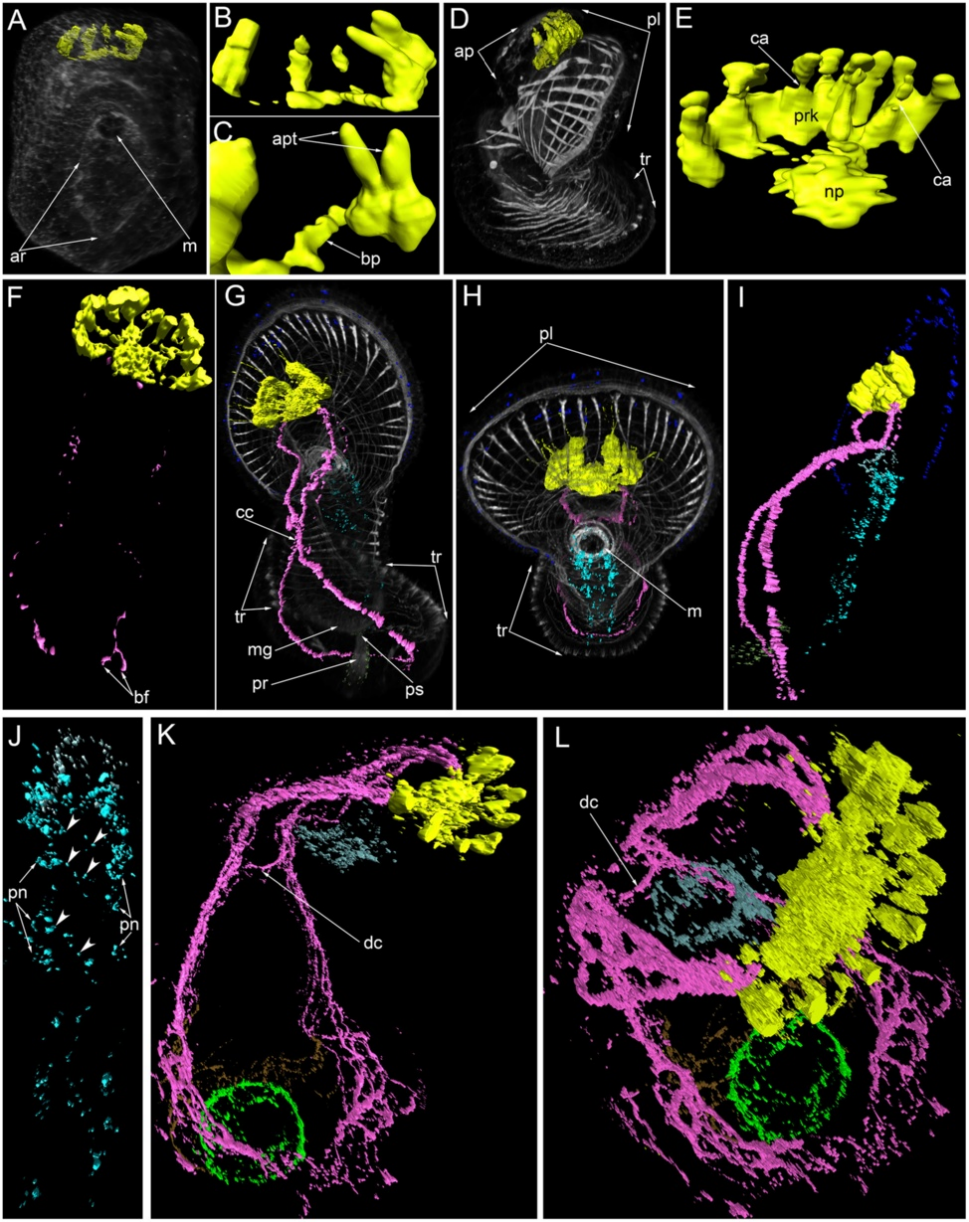
**Three-dimensional reconstructions of the serotonin-like immunoreactive nervous system in consecutive stages of development.** Color: yellow – apical organ; pink – tentacular neurite bundle; light green – telotroch neurites; dark green – nerve net around the proctodaeum; cyan – ventral nerve cord; pale blue – oral nerve ring; dark blue – marginal neurites of the preoral lobe; brown – trunk neurites; grey/white – muscle system and all actin-containing structures (parietal cytoplasm, microvilliy, etc.). (**A**) Mid-gastrula, dorsal view, apical is to the top. The mouth (m) and archenteron (ar) are visible. (**B**) Dorsal view of the apical organ which consists of four or five perikarya and their neurites. (**C**) Two adjacent perikarya which have two apical parts (apt) and one basal process (bp). (**D**) Lateral view of a preactinotrocha (ventral side to the right) showing apical plate (ap), preoral lobe, and tentacular ridge (tr). (**E**) Dorsal view of the apical organ in a preactinotrocha showing perikarya (prk), neuropil (np), and constricted areas (ca) between the apical and basal parts of some perikarya. (**F**) Dorsal view of the nervous system in a young actinotrocha, which has a large apical organ and two thin dorso-lateral branches of the tentacular neurite bundles. The branches bifurcate (bf) at the terminal end. (**G**) Dorso-lateral view of a 6-day-old actinotrocha (preoral lobe bends backward) showing midgut (mg), proctodaeum (pr), pyloric sphincter (ps), and tentacular ridge (tr). The two dorsal branches of the tentacular neurite bundle form close contact (cc) on the dorsal side. (**H**) The same larva, anterior view. The tentacular ridge contains latero-frontal cells, which have long thick microvilli strongly stained by phalloidin. The mouth is marked by thick muscles; the preoral lobe (pl) bends backward. (**I**) The same larva, lateral view of the nervous system. (**J**) The same larva; the image includes only the ventral part of the nervous system of the oral field. The paired ventral neurite bundle contains paired perikarya (pn) and thin commissures (arrowheads). (**K**) Ventro-lateral view of the nervous system in a 24-day-old larva. The tentacular neurite bundle is very thick and forms several loops in each tentacle. The two dorsal branches of the tentacular neurite bundle interconnect via a dorsal commissure (dc). (**L**) The same larva, ventro-lateral top view. Ring-shaped neurites around the mouth and the telotroch are visible.

In the late gastrula I (Figure 2E), the basal processes of serotonin-like immunoreactive perikarya form a neuropil in the center of the apical plate (Figure 2F). Nerve fibers occupy the basal portion of the apical plate and are in close contact with the mesodermal cells (Figure 2F). At this stage, the shape of perikarya does not change but the number increases with age and is seven or nine in the late gastrula II (Figure 2G). The arrangement of the perikarya forms a horseshoe-like pattern along the anterior edge of the apical plate. The apical portion of the cell becomes longer (Figure 2J). The neuropil becomes rounded and larger (Figure 2J). At this stage, and usually within the apical organ, there are two perikarya which are situated very closely adjacent to each other and form a cluster with two apical parts, two nuclei, and one process (Figures 2H, 3C).

The number of perikarya in the apical organ increases and the preactinotrocha has 12 perikarya (Figures 2K, L, 3D, E). The apical organ also increases in size and the perikarya are arranged in a wide horseshoe-like pattern with a large neuropil in the center (Figure 3D, E).

**Figure 4:**
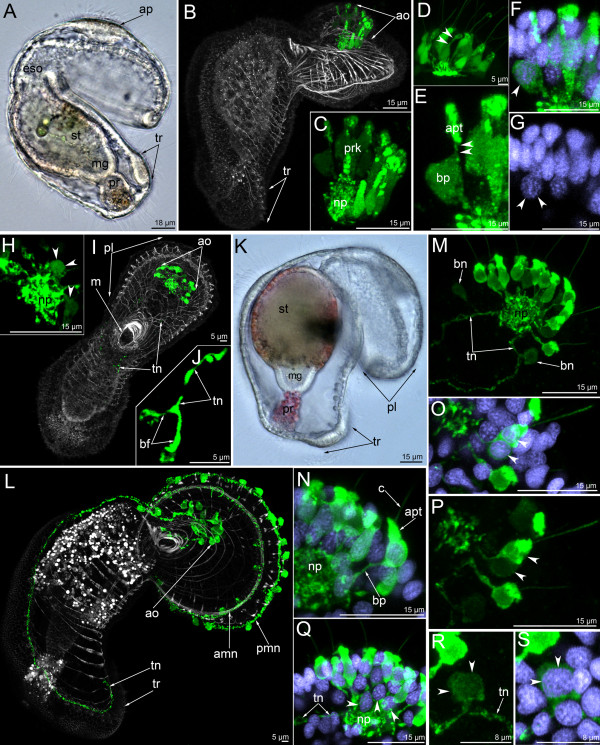
**The organization of the serotonin-like immunoreactive nervous system in a young actinotrocha (A-I) and in a 5-day-old actinotrocha (K-S).** Micrographs of live animals (A, K) and Z-projections of larvae with mono- and double staining with antibodies against 5-HT (serotonin) (green),as well as by phalloidin (grey), and Hoechst (violet). (**A**) Lateral view of a live larva (ventral side is to the right, apical is to the top) showing apical plate (ap), esophagus (eso), stomach (st), midgut (mg), proctodaeum (pr), and tentacular ridge (tr). (**B**) Overview of the musculature and serotonin-like immunoreactive nervous system showing apical organ (ao) and tentacular ridge. Lateral view, ventral side is to the right, apical is at the top. (C-H) Details of the apical organ. (**C**) General view of the apical organ with neuropil (np) and perikarya (prk). Lateral view, apical is to the top; anterior is to the right. (**D**) Dorsal view of the apical organ. The thin area between apical and basal parts of the perikaryon is indicated by two arrowheads. (**E**) Perikaryon with pronounced thin area (two arrowheads) between apical (apt) and basal (bp) parts. (**F**) The same perikaryon with nucleus. Wide basal part is indicated by arrowhead. (**G**) The same perikaryon nucleus (arrowheads), which is located under the common row of nuclei. (**H**) Basal portion of the apical organ showing neuropil and bipolar or multipolar (arrowheads) perikarya. (**I**) Young larva with the first serotonin-like immunoreactive neurites that subsequently form the two dorsal branches of the tentacular neurite bundle (tn). Overview of the musculature and serotonin-like immunoreactive nervous system showing apical organ (ao), mouth (m), and preoral lobe (pl). Ventral view, preoral lobe bends backward. (**J**) The same larva; bifurcated (bf) end of left branch of the tentacular neurite bundle. The micrograph is composed of selected optical sections from the dorsal-most region of the specimen. (**K**) Lateral view of a 5-day-old larva showing preoral lobe, stomach, midgut, and proctodaeum. Apical is to the upper right. (**L**) Overview of the musculature and serotonin-like immunoreactive nervous system showing apical organ, anterior (amn) and posterior (pmn) marginal neurite bundles, tentacular neurite bundle (tn), and tentacular ridge (tr). Lateral view; preoral lobe bends backward; apical is to the upper right. Digestive tract with nonspecific staining (numerous white vesicles in the stomach and proctodaeum). (M-S) Details of the apical organ. (**M**) General view of the apical organ showing neuropil (np), bipolar perikarya (bn), and two dorsal branches of the tentacular neurite bundle (tn). Dorso-lateral view; anterior is to the upper right. (**N**) General view of an individual monopolar perikaryon showing cilium (c), apical part (ap), and basal projection (bp), which connects to the neuropil (np). (**O**) Two serotonin-like immunoreactive perikarya, which are adjacent to each other and form a cluster with two apical parts, two nuclei (arrowheads), and one process. (**P**) The same perikarya. (**Q**) The group of bipolar or multipolar perikarya, nuclei (arrowheads) of which are located close to the neuropil. (**R**) Bipolar perikaryon (arrowheads), which is located on the one branch of the tentacular neurite bundle. (**S**) The same perikaryon with nucleus (arrowheads).

**Figure 5:**
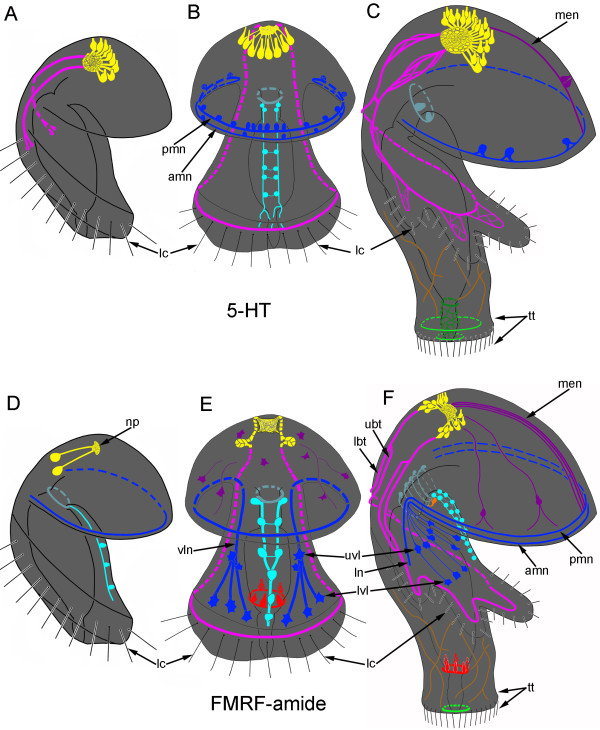
**Schemes of the development of serotonin-like (A-C) and FMRFamide-like (D-F) immunoreactive nervous system in *Phoronopsis harmeri* .** (**A**, **D**) Young actinotrocha. (**B**, **E**) 6-day-old actinotroch larva. (**C**, **F**) 24-day-old actinotroch larva. Color code: yellow – apical organ; pink – tentacular neurite bundles; light green – telotroch neurites; dark green – nerve net and circular neurites around the proctodaeum; cyan – ventral neurite bundles; pale blue – oral nerve ring; dark blue – marginal neurite bundles of the preoral lobe edge, lateral (ln) and ventro-lateral (vln) neurites of the oral field, upper ventro-lateral perikarya (uvl), and lower ventro-lateral perikarya (lvl); brown – trunk neurites; magenta – median neurite bundle (men) with perikarya, neurites, and perikarya of the preoral lobe; orange – large perikarya near the mouth (lp). Abbreviations: lc – motionless long cilia along the tentacular ridge; np – neuropile; t – tentacle; tn – trunk; tr – tentacular ridge; tt – telotroch with cilia.

In young actinotrochs (Figure 4A), the apical organ retains its previous organization and consists of 13–15 monopolar, monociliated flask-shaped cells (Figure 4B, C). At the same time, the shape of some perikarya starts to change (Figure 4D-F). The apical organ contains one or two perikarya, which have a thin area between the nucleus and the apical part of the cell (Figure 4E). This area becomes progressively thinner, whereas the basal part of the cell (the part with the nucleus) becomes wider (Figure 4E). Simultaneously, the basal part of the cell migrates to the basal portion of the apical plate, and the nucleus of the perikaryon is then located under the common row of nuclei (Figure 4G). Perikarya with thin median portions are best identified in three-dimensional reconstructions (Figure 3E). The bipolar or multipolar perikarya result from these processes. Later, these bipolar perikarya can be found within the perikarya of the apical organ (Figure 4H). The young actinotroch usually has two or three bipolar perikarya within the apical organ. They are located in front of the neuropil and on the dorso-lateral side of the neuropil. The first serotonin-like immunoreactive neurites appear in the young actinotroch (Figures 3F, 4I, 5A). The pair of dorso-lateral neurites originates from the neuropil and passes along the dorso-lateral sides of the preoral lobe to the dorsal ends of the postoral ciliated band (Figures 4I, 5A). Here, each nerve tract bifurcates into short branches with swollen ends (Figures 3F, 4J, 5A).

In the 5-day-old actinotroch (Figure 4K), additional serotonin-like immunoreactive neurites appear along the edge of the preoral lobe. They form two rows, which constitute the anterior and posterior marginal neurite bundles (Figure 4L). Each anterior marginal nerve passes along the marginal muscle of the preoral lobe and contains 4–5 bipolar serotonin-like immunoreactive perikarya. The posterior marginal neurite bundle is stained more intensely than the anterior marginal neurite bundle and connects with 12–15 large serotonin-like immunoreactive perikarya (Figure 4L). The anterior and posterior margin neurite bundles merge at the dorsal ends of the preoral lobe. Dorso-lateral neurite bundles emerging from the neuropil increase in diameter, pass along the tentacular ring, and form contact on the ventral side, forming the tentacular nerve ring (Figure 4L). The number of monopolar perikarya within the apical organ does not increase, but the shape of the perikarya does (Figure 4M). In the 5-day-old actinotroch, monopolar perikarya have a long basal process, which can reach 6 μm in length. The basal part of the perikaryon is wide and rounded (Figure 4N) and the nucleus is located here. The apical part of the cell resembles a collar and stains intensely. A serotonin-like immunoreactive cell with one basal process, two rounded nuclei (basal and apical), a small apical part, and a cilium (Figure 4O, P) can be found among other perikarya. Within the apical organ, there are bipolar or multipolar perikarya, which are located among the fibers of the neuropil (Figure 4Q); these perikarya have a large and irregularly shaped nucleus and they always occupy the anterior and lateral edge of the neuropil (Figure 4Q). Two bipolar cells can usually be found near the apical organ (Figure 4M). These cells are associated with the dorso-lateral branches of the tentacular neurite bundle and are located on both sides of the apical organ. A round nucleus is located in the center of the perikaryon.

**Figure 6:**
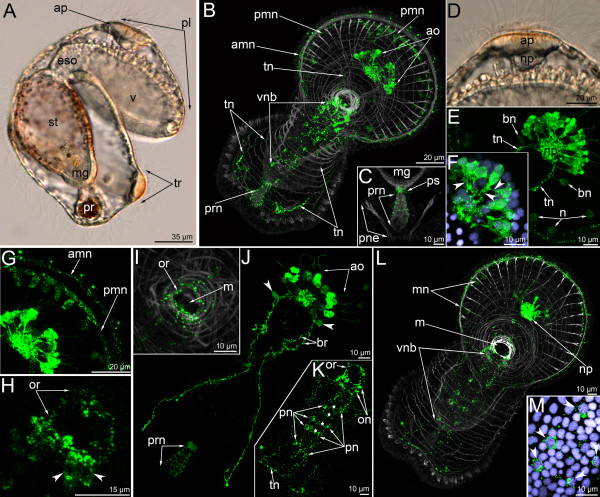
**The organization of the serotonin-like immunoreactive nervous system in a 6-day-old larva.** Micrographs of live animals (**A**) and Z-projections of larvae after mono- and double staining with antibodies against serotonin (green), as well as with phalloidin (grey), and Hoechst (violet). Apical is to the upper right on all micrographs except A, C, and D, where apical is to the top. (A) Lateral view with ventral to the right. Larva with apical plate (ap), preoral lobe (pl), vestibulum (v), esophagus (eso), stomach (st), midgut (mg), proctodaeum (pr), and tentacular ridge (tr). (**B**) Overview of the musculature and serotonin-like immunoreactive nervous system showing apical organ (ao), anterior marginal neurite bundle (amn), posterior marginal neurite bundle (pmn), tentacular neurite bundle (tn), and ventral neurite bundles (vnb). Ventral view; preoral lobe bends backward. (**C**) Posterior body part. The micrograph comprises selected optical sections from the mid body region of the specimen and shows the nerve net around the proctodaeum (prn) and the pyloric sphincter (ps), midgut (mg), and protonephridia. (**D**) Lateral view of the apical plate (ap) and large neuropile (np) underneath. The anterior pole of the preoral lobe is to the right. (**E**) Top view of the apical organ with dorsal neuropil (np), dorso-lateral branches of the tentacular neurite bundle (tn), and bipolar perikarya (bn). Some portions of the marginal neurites with perikarya (n) are visible. (**F**) Details of the apical organ, anastomosing neurites are indicated by arrowheads. (**G**) Anterior portion of the edge of the preoral lobe with anterior (amn) and posterior (pmn) marginal neurite bundles with a group of perikarya associated with the latter. (**H**) Oral nerve ring with ventro-lateral, weakly stained perikarya (arrowheads); top view. (**I**) Innervation (oral nerve ring – or) and musculature of the mouth (m); ventral view. (**J**) Dorsal portion of the tentacular neurite bundle (tn) with two branches (br) of the right stem. apical organ (ao), bipolar perikarya (arrowheads), and nerve net of the proctodaeum (prn). (**K**) Oral field with the ventral neurite bundles, which pass from the oral ring (or) with oral perikarya (on) to the tentacular neurite bundle (tn), containing several paired perikarya (pn) and commissures (asterisks). The micrograph is composed of the most ventral optical sections only. (**L**) The image comprises only the four most ventral optical sections of the serotonin labeling and all sections of the muscle staining. Ventral view of a larva showing the marginal neurites (mn) of the preoral lobe, the neuropil (np) of the apical organ, the mouth (m), and the ventral neurite bundles (vnb). (**M**) Some paired perikarya of the ventral neurite bundles with nuclei (arrowheads).

In the 6-day-old actinotroch larva (Figure 6A), the serotonin-like immunoreactive nervous system becomes more complex (Figures 3G-J, 5B, 6). All previous elements are retained and new elements appear (Figure 6B). At this stage, typically, the apical organ contains 12–15 monopolar serotonin-like immunoreactive perikarya, but up to 25 monopolar perikarya were found within the apical organ in some larvae (Figure 6E). The neuropil of the apical organ becomes more elaborate and can be recognized even in live larvae, where it resembles a transparent bubble under the apical plate epithelium (Figure 6D). The basal processes of the monopolar perikarya branch before they form contact with the neuropil and form a neural meshwork (Figure 6F). Bipolar or multipolar perikarya occur in the neuropil. The posterior and anterior marginal neurite bundles of the preoral lobe retain several bipolar perikarya. These neurites can not be recognized in subsequent stages (Figure 6B, E). Approximately 14 perikarya are distributed along the anterior marginal neurite bundles and five of them are concentrated in its median portion (Figure 6G). The dorso-lateral neurite bundle of the tentacular ring divides into two near the neuropil (Figure 6J). The branches of one neurite bundle fuse on the dorsal side. Three-dimensional reconstruction reveals that the tentacular neurite bundle runs along the lower edge of the tentacular ridge. As in the previous stage, two bipolar perikarya are associated with the two dorso-lateral neurite bundles. These perikarya are located on both sides of the neuropil (Figure 6E, J). In the 6-day-old larva, an oral nerve ring appears (Figure 6H, I, K). It is composed of thin circular neurites, which also extend along the esophagus. The oral nerve ring does not contact the apical organ. In some larvae, serotonin-like immunoreactive perikarya occur under the oral ring and on its left and right sides (Figure 6H). Larvae of this stage also have serotonin-like immunoreactive processes around the proctodeum and the pyloric sphincter (Figure 6C, J). Interestingly, immunofluorescence can be observed in the apical portion of the epithelium of the proctodaeum (Figure 6C). A paired serotonin-like immunoreactive neurite bundle appears in the epithelium of the oral field, between the oral nerve ring and the tentacular neurite bundle (Figure 6B, K, L). It consists of two longitudinal neurite bundles and commissures, which interconnect the perikarya of the two neurite bundles. Each longitudinal neurite bundle includes thin neurites and six perikarya and contacts the oral nerve ring via paired perikarya, which are situated in close proximity to the oral nerve ring (Figure 5B, 6K, L). This serotonin-like immunoreactive paired ventral neurite bundle was previously described [22], and here we provide micrographs of a different larval specimen (Figure 6K-M). 3D reconstruction of another larva reveals the presence of repetitive perikarya and thin commissures between them (Figure 3H, J).

**Figure 7:**
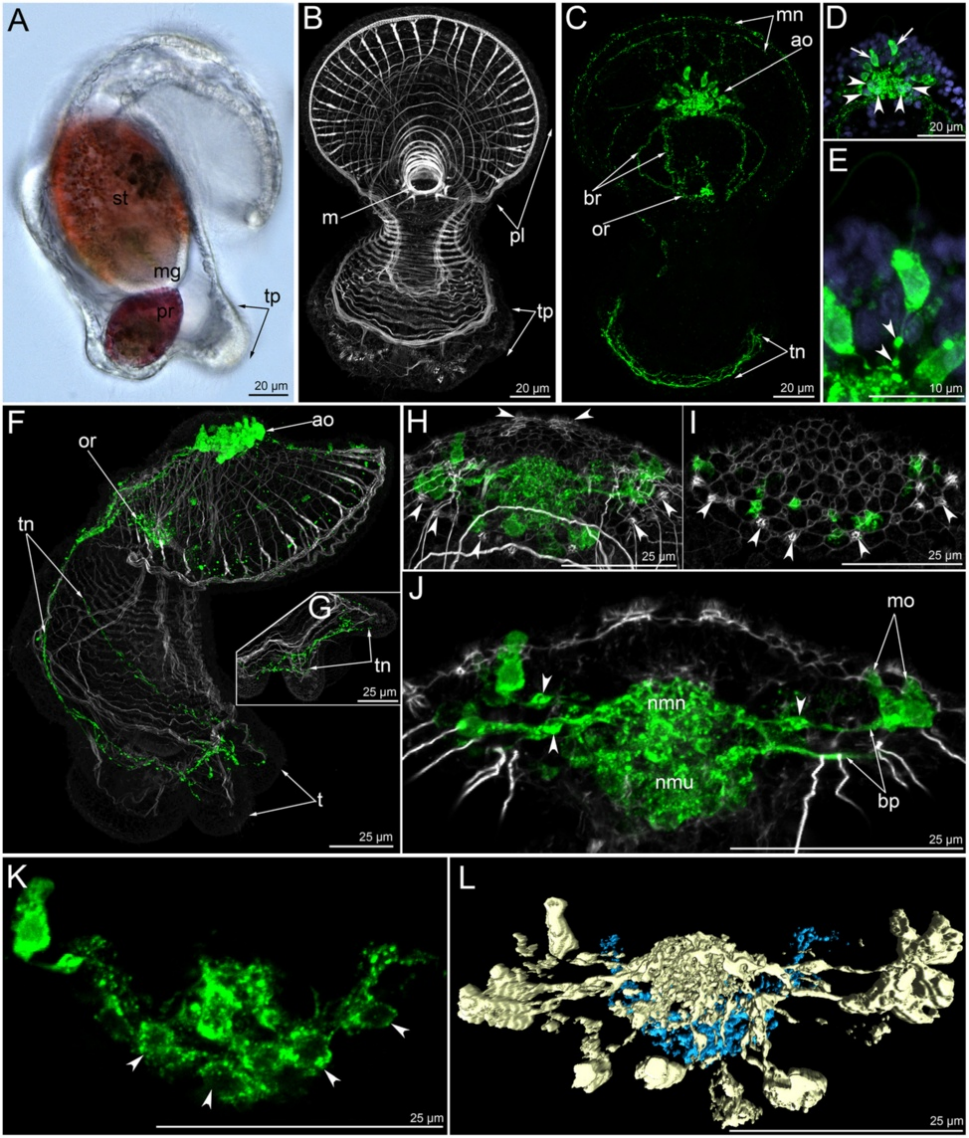
**The organization of the serotonin-like immunoreactive nervous system in a 13-day-old larva.** Micrographs of a live animal (A), Z-projections (B-K) of larvae after mono- and double staining with antibodies against 5-HT (serotonin) (green), as well as staining with phalloidin (grey), and Hoechst (violet), and 3-D reconstruction (L). Apical is to the top in all aspects. (**A**) Lateral view of live larva showing stomach (st), midgut (mg), proctodaeum (pr), and primordia of tentacles (tp). (**B**) Overview of the muscle system; ventral view; preoral lobe bends backward. General view showing preoral lobe (pl), mouth (m), and primordia of tentacles (tp). (**C**) The same larva; overview of the serotonin-likeimmunoreactive system; ventral view showing weakly stained marginal nerves, apical organ (ao), branches (br) of the dorsal portion of the tentacular neurite bundle, oral ring (or), and ventral portion of the tentacular neurite bundle (tn). (D-E) Details of the apical organ; anterior view. (**D**) General view of the apical organ, which is composed of monopolar (arrows) and bipolar or multipolar (arrowheads) perikarya. (**E**) Monopolar perikaryon with varicosities (arrowheads) on the basal process. (**F**) Overview of the musculature and the serotonin-like immunoreactive nervous system showing apical organ (ao), oral ring (or), tentacular neurite bundle (tn), and tentacles (t). Lateral view; ventral is to the right. (**G**) Organization of the ventral portion of the tentacular neurite bundle (tn) which forms a loop in each tentacle. (**H**) Ventral view of the apical plate with apical organ and rows of sensory cells (arrowheads). (**I**) The micrograph comprises the three ventral-most optical sections of the confocal stack, i.e., the region of the mutual position of monopolar perikarya and sensory cells (arrowheads). (**J**) Frontal view of the center of the apical organ showing monopolar perikarya (mo), their basal processes (bp) with varicosities (arrowheads) and neuropil (nmn), and bipolar or multipolar perikarya and neuropil (nmu). (**K**) Organization of bipolar or multipolar perikarya (arrowheads); ventral view. (**L**) Three-dimensional reconstruction of the apical organ. Light blue indicates multipolar or bipolar perikarya, which form a horseshoe-shaped row under the neuropil of the monopolar perikarya (golden); ventral view.

In 13-day-old actinotrochs, the general organization of the nervous system changes (Figure 7A-C). The anterior and posterior marginal neurite bundles become very weak, and serotonin-like immunoreactive perikarya along them are not evident (Figure 7C). The paired ventral neurite bundle can not be recognized. The tentacular neurite bundle becomes more complex. Its dorso-lateral parts bifurcate and each branch forms two additional branches, which fuse on the dorsal side (Figure 7C). The ventral portion of the tentacular neurite bundle also changes such that many thin small loops extend along its edge (Figure 7C). The apical organ contains numerous bipolar or multipolar perikarya (Figure 7D). The number of monopolar perikarya does not increase substantially. The basal processes of the monopolar perikarya bear several varicosities (Figure 7E). When the tentacles become more pronounced, branches of the tentacular neurite bundle extend into each tentacle (Figure 7F). A neurite bundle runs along the abfrontal side of each tentacle (Figure 7G). The apical plate has a complex structure and contains sensory cells and perikarya (Figure 7H). Sensory cells can be recognized by the presence of long microvilli on the apical surface; these microvilli are strongly stained by phalloidin (Figure 7H, I). Sensory cells form an external row on the periphery of the apical plate and form two rows in the center. Double staining with phalloidin and against serotonin reveals the position of serotonin-like immunoreactive perikarya and sensory cells (Figure 7I). The former are situated on the periphery of the apical plate between the sensory cells. Thus, serotonin-like immunoreactive perikarya are distant from the center of the apical plate where the neuropil is situated. For this reason, each monopolar perikaryon forms a long (16 μm) basal process (Figure 7J). Large varicosities occur along the process. Bipolar or multipolar perikarya form a layer under the neuropil, along its anterior edge (Figure 7K, L). This layer is composed of 8–10 perikarya. The most dorsal multipolar perikarya give rise to the dorso-lateral branches of the tentacular neurite bundle (Figure 7L).

**Figure 8:**
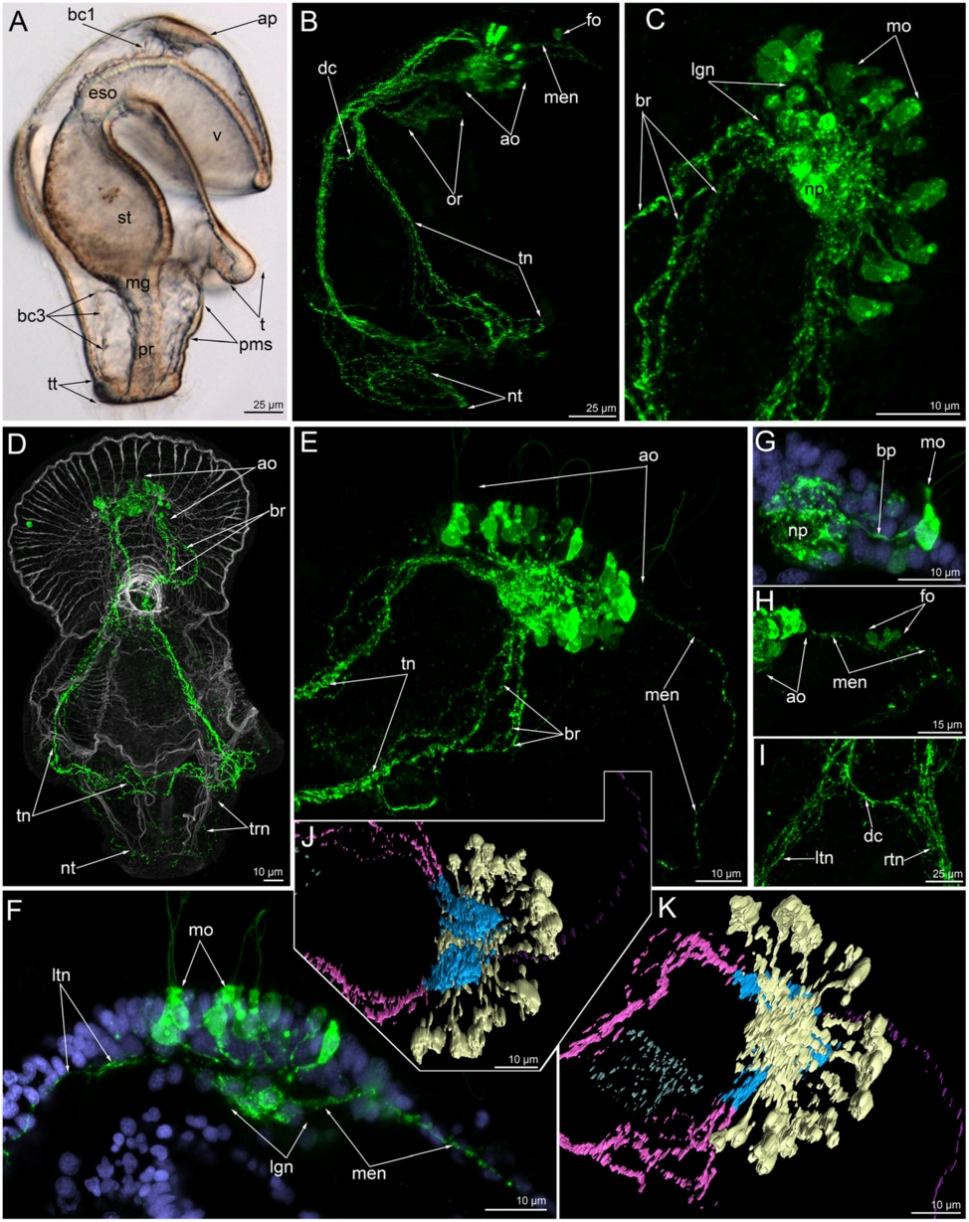
**Organization of the serotonin-like immunoreactive nervous system in a 24-day-old larva.** Micrographs of a live animal (A), Z-projections (B-I) of larvae after mono- and double staining with antibodies against 5-HT (serotonin) (green), as well as staining with phalloidin (grey), and Hoechst (violet), and 3-D reconstructions (J-K). (**A**) General view of a live larva showing the apical plate (ap), the border of the preoral coelom (bc1), vestibulum (v), esophagus (eso), stomach (st), midgut (mg), proctodaeum (pr), the border of the trunk coelom (bc3), tentacle (t), telotroch (tt), and primordium of the metasomal sac (pms). Lateral view; apical is to the top; ventral is to the right. (**B**) Nervous system with all major elements: apical organ (ao), median neurite bundle (men), frontal organ (fo), oral nerve ring (or), tentacular neurite bundle (tn) with dorsal commissure (dc), and nerve ring of the telotroch (nt). Lateral view; apical is to the top, ventral to the right. (**C**) Dorsal view of branched proximal ends of the tentacular neurite bundle (br) and apical organ, which is composed of monopolar (mo) and bipolar or multipolar perikarya. The latter forms the larger group on the left (lgn), which rises above the neuropil (np). Anterior is to the upper right. (**D**) Overview of the musculature and serotonin-like immunoreactive nervous system showing apical organ (ao), tentacular neurite bundle (tn) which branches (br) on the dorsal side, thin trunk neurites (trn), and nerve ring of the telotroch (nt). Ventral view; apical is to the top; preoral lobe bends backward. (**E**) Dorso-lateral view of the preoral lobe showing apical organ (ao), median neurite bundle (men), and branches (br) of the tentacular neurite bundle (tn). Anterior is to the right. (**F**) Left portion of the apical organ showing monopolar perikarya (mo) and the left group of bipolar or multipolar perikarya (lgn), which gives rise to the left branch of the tentacular neurite bundle (ltn) and median neurite bundle (mn) of the preoral lobe. Lateral view; apical is to the top; anterior of the preoral lobe is to the right. (**G**) Details of a monopolar perikaryon; lateral view showing perikaryon (mo) and basal projection (bp) that passes from the median part of the cell to the neuropil (np). (**H**) Lateral view of the preoral lobe showing anterior part of the apical organ (ao), median neurite bundle (mn), and frontal organ (fo), which is composed of several weakly stained perikarya. Anterior of the preoral lobe is to the right. (**I**) Dorsal commissure (dc) between two dorso-lateral branches of left (ltn) and right (rtn) branches of the tentacular neurite bundle. Dorsal view; apical is to the top. (**J**) Posterior view of the apical organ that is composed of two groups of bipolar or multipolar perikarya (light blue), which give rise to two dorso-lateral branches of the tentacular neurite bundle (light pink) and median neurite bundle (magenta). Monopolar perikarya form the upper large neuropil (golden). Anterior is to the right; left is to the bottom. (**K**) Top view of the apical organ showing large neuropil and monopolar perikarya (golden), neuropil and bipolar or multipolar perikarya (light blue), tentacular neurite bundle (light pink), median neurite bundle (magenta), and oral nerve ring (pale blue).

In 24-day-old actinotrochs the general organization of the serotonin-like immunoreactive nervous system changes (Figure 5C, 8). At this stage, the apical organ and the tentacular neurite bundle are the main elements of the nervous system (Figure 8B, D). The apical organ is composed of 20–25 monopolar perikarya and 12–15 bipolar or multipolar perikarya (Figure 8C, E). Each monopolar perikaryon has a flask-like shape with a wide basal part and a narrow apical part (Figure 8F). The basal part of the cell forms a single process, which usually arises from the terminal basal part of the perikaryon. In some perikarya, however, it projects from the central part of the perikaryon (Figure 8G). The processes of all monopolar perikarya extend to the center of the apical organ and contribute to the apical neuropil (Figure 8K). Under this neuropil, bipolar or multipolar perikarya and their processes are situated (Figure 8J, K). They are arranged in two groups, left and right, and each group consists of 4–5 spherical perikarya that are 5–6 μm in diameter (Figure 8F). Interestingly, in some larvae the left dorso-lateral group of multipolar perikarya form a large mass, which rises above the neuropil (Figure 8C). The multipolar perikarya are located near the anterior and lateral edge of the neuropil, whereas their processes occupy the dorsal portion. The serotonin-like immunoreactive part of the neuropil is ovoid and 16–17 μm long and 8–9 μm wide.

The 24-day-old actinotroch has a weak marginal neurite bundle with several perikarya (Figure 5C) and a prominent median neurite bundle, which extends from the apical organ to the edge of the preoral lobe along its median line (Figure 8E, H, J, K). The median neurite bundle arises from the left group of multipolar perikarya (Figure 8F, J). In some larvae, a group of weakly stained perikarya is found in the center of the median neurite bundle (Figure 8H). This group consists of three small perikarya that contribute to the frontal organ. In some larvae, there is only one perikaryon instead of three (Figure 8B). Two dorso-lateral branches of the tentacular neurite bundle arise from the dorso-lateral parts of the neuropil where the multipolar perikarya are situated (Figure 8J, K). Near the apical organ, each dorso-lateral neurite bundle branches into several neurite bundles, which fuse on the dorsal side (Figure 8B, E). Here, the dorso-lateral neurite bundles are connected to each other via a dorsal commissure (Figure 8B, I). Along the whole length, the tentacular neurite bundle is formed by 7–10 neurites (Figure 8I). The tentacular neurite bundle runs under the tentacular ridge and forms a loop along the abfrontal side of each tentacle. The oral nerve ring is composed of thin circular neurites and several perikarya, which usually stain weakly (Figure 8B). These perikarya are located in the oral field epithelium, under the mouth. The oral nerve ring does not contact the tentacular neurite bundle or the apical organ, as stated previously [23]. Thin neurites form a cylinder, which matches the shape of the esophagus (Figures 3K, 8B). In the 24-day-old larva, the trunk and the telotroch have formed (Figures 3L8B). The telotroch is innervated by two serotonin-like immunoreactive neurite bundles: the inner and the external one. They form two circles in the posterior end of the body (Figure 3L). Thin nervous fibers pass along the lateral and dorsal sides of the larval trunk (Figure 3K).

#### FMRFamide-like immunoreactive nervous system

**Figure 9:**
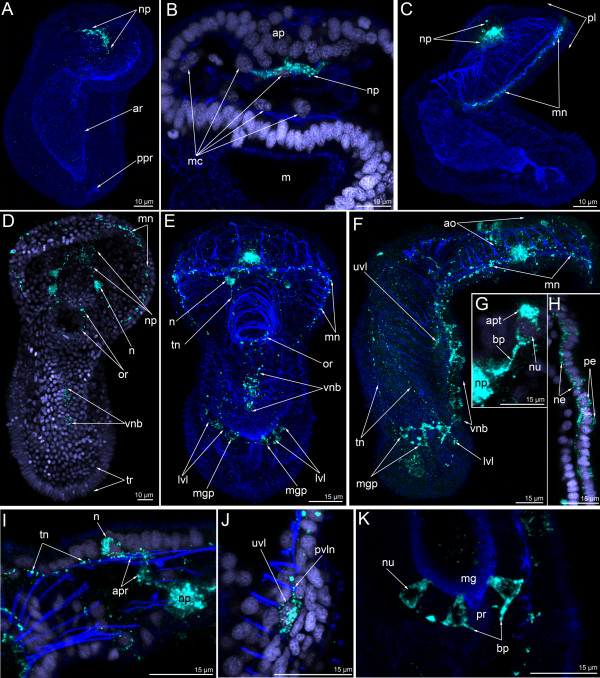
**Organization of the FMRFamide-like immunoreactive nervous system in the late gastrula (A, B), preactinotrocha (C), and young larva (D-K).** Z-projections of larvae after double and triple staining with antibodies against FMRFamide (cyan), as well as staining with phalloidin (blue), and Hoechst (violet). Apical is to the top in all cases except C, where it is to the upper left. (**A**) Overview showing apical organ, which is represented by a horseshoe-shaped neuropil (np), archenteron (ar), and precursor of the proctodaeum (ppr). Dorso-lateral view. (**B**) Median region of the anterior body part showing apical plate (ap) with neuropil (np), mesodermal cells (mc), and mouth (m); ventral view. (**C**) Overview of a preactinotrocha with large apical neuropil (np) and marginal neurite bundle (mn) running along edge of the preoral lobe (pl). Lateral view; ventral side is to the right. (**D**) Overview of nuclei and FMRFamide-like immunoreactive nervous system showing marginal neurite bundle (mn), neuropil (np), first perikarya (n) of the apical organ, oral nerve ring (or), first neurites of the ventral neurite bundle (vnb), and tentacular ridge (tr). Ventral view. (**E**) Overview of the musculature and FMRFamide-like immunoreactive nervous system showing marginal neurite bundle (mn), perikarya (n) of the apical organ, the tentacular neurite bundle (tn), oral ring (or), lower ventro-lateral perikarya (lvl) of the oral field, perikarya associated with the ventral neurite bundle (vnb), and perikarya associated with the midgut (mgp). Ventral view. (**F**) Lateral view of a young larva with apical organ (ao), marginal neurite bundle (mn), thin tentacular neurite bundle (tn), upper ventro-lateral perikaryon (uvl), lower ventro-lateral perikaryon (lvl), perikarya of the midgut (mgp), and perikarya of the ventral neurite bundles (vnb). Lateral view; ventral side is to the right. (**G**) Monopolar perikaryon of the apical organ with bright apical cell part (apt), nucleus (nu), and basal process (bp) projecting to the neuropil (np). (**H**) Ventral body wall of a larva with perikarya (pe) and neurites (ne) of the ventral neurite bundles. (**I**) Bipolar perikaryon of the apical organ with two processes: anterior process (apr), which runs into the neuropil (np), and posterior process (pth), which is the precursor of one stem of the tentacular neurite bundle. Lateral view; anterior is to the right. (**J**) Ventro-lateral body wall with large upper ventro-lateral perikaryon (uvl) and the ventro-lateral neurite (pvln). Lateral view; ventral is to the right. (**K**) Posterior body part with midgut (mg), proctodaeum (pr), and perikarya of the midgut, which contain the non-stained nuclei (nu) and form basal processes (bp). Lateral view; ventral is to the right.

**Figure 10:**
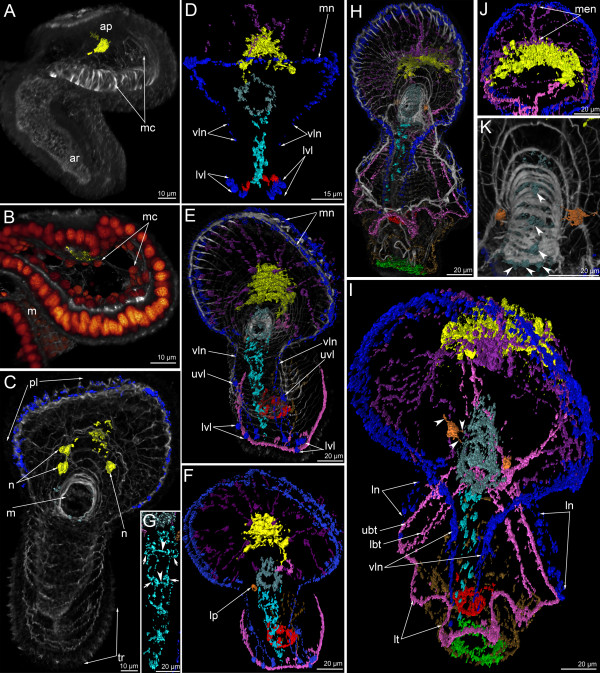
**Three-dimensional reconstructions of the FMRFamide-like immunoreactive nervous system in consecutive stages of development.** Color: yellow – apical organ; pink – tentacular neurites; light green – telotroch neurites; cyan – ventral neurite bundle; pale blue – oral nerve ring; dark blue – marginal neurites of the preoral lobe edge, lateral (lt) and ventro-lateral (vln) neurites of the oral field, upper ventro-lateral perikarya, and lower ventro-lateral perikarya; brown – trunk neurites; magenta – neurites and perikarya of the preoral lobe; orange – large perikarya near the mouth (lp); red – circular neurites circle with perikarya around the midgut; reddish-orange – nuclei; grey/white – muscle system and all actin-containing structures (parietal cytoplasm, microvilli, etc.). Apical is to the top in all cases. (**A**) General view of a late gastrula showing apical plate (ap), archenteron (ar), and mesodermal cells (mc). Lateral view; ventral is to the right. (**B**) Anterior portion of the body of a late gastrula. Close contact between the neuropil of the apical organ (yellow) and mesodermal cells (mc) is evident. Sagittal optical section through the mouth (m); ventral is to the right. (**C**) Young actinotrocha; overview of the musculature and the FMRFamide-like immunoreactive nervous system showing the first perikarya (n) of the apical organ, the preoral lobe (pl) with marginal neurite bundle, the mouth with the oral nerve ring (or), and the tentacular ridge (tr). Ventral view. (**D**) Organization of the nervous system in the young actinotrocha. At this stage, the ventro-lateral neurites (vln) of the oral field, the marginal neurite bundle (mn) of the preoral lobe, the lower ventro-lateral perikarya (lvl), and the midgut perikarya are present. (**E**) Overview of the musculature and the nervous system in a 6-day-old larva; ventro-lateral view. The stack, which was used to produce the micrographs, lacks the most dorsal optical sections and the tentacular neurite bundle is not visible in the most dorsal region. The marginal neurite bundle (mn) of the preoral lobe continues towards the oral field and passes into the ventro-lateral neurites (lvn), which connect with the upper ventro-lateral perikarya (uvl), and the lower ventro-lateral perikarya (lvl). (**F**) Nervous system of a 6-day-old larva. (**G**) Ventral line of the oral field with ventral neurite bundles with associated paired perikarya (arrows) and commissures (arrowheads). (**H**) Overview of the musculature and nervous system in a 24-day-old larva; ventro-lateral view. (**I**) Organization of the FMRFamide-like immunoreactive nervous system in a 24-day-old larva; ventro-lateral view showing lateral neurites (ln), ventro-lateral neurites (vln), the upper branch of the tentacular neurite bundle (ubt), the lower branch of the tentacular neurite bundle (lbt), and the loops of the tentacle neurites (lt). Neurites of a large perikaryon near the mouth are indicated by arrowheads. (**J**) Dorsal view of the preoral lobe with large apical organ and radial neurites including the prominent median neurite bundle (men). (**K**) Dorsal view of the esophageal musculature and the nervous system (perikarya are indicated by arrowheads).

The earliest signal of the neuropeptide FMRFamide is found in the late gastrula and is located in the apical plate (Figures 9A, 10A). Cell processes are labeled at this stage, but no cell bodies were found (Figure 9B). Labeled cell processes form a neuropil in the center of the basal portion of the apical plate (Figure 9B). Mesodermal cells forming the protocoel lining contact the neurites of the FMRFamide-immunoreactive neuropil (Figure 10B). Three-dimensional reconstruction reveals that the neuropil is shaped like a horseshoe with two dorso-lateral branches (Figure 10A).

In the preactinotroch, the thin and interrupted FMRFamide-immunoreactive neurites appear along the edge of the preoral lobe (Figure 9C). Interestingly, the first FMRFamide reactive neurite runs along the edge of the preoral lobe, whereas the serotonin-like immunoreactive neurite extends from the apical organ to the tentacular ridge.

The first perikarya with FMRFamide-like immunoreactivity occur in the young actinotroch. Perikarya are situated on the dorso-lateral side of the preoral lobe behind the neuropil (Figures 5D, 9D, 10C). Usually, two or three perikarya appear simultaneously. These are large bipolar cells, which connect to the neuropil via thin neurites (Figure 9D). In young actinotrochs, several thin neurites originate around the mouth and along the medio-ventral line of the oral field (Figure 9D). These neurites are very weak (Figure 10C).

After several hours, new FMRFamide-immunoreactive elements appear in young actinotrochs (Figure 9E, F). These are the perikarya of the oral field epidermis, perikarya in the midgut, and radial neurites in the preoral lobe (Figures 9E-K, 10D). In young actinotroch larvae, the apical organ consists of bipolar and monopolar perikarya. The latter is column-shaped with wide apical and basal parts (Figure 9G). The basal part contains the nucleus; the apical part is always brightly stained. The basal part of the perikaryon forms a process that passes into the neuropil. Bipolar perikarya are located distant from the neuropil and connect to it via long anterior processes (Figure 9I). The posterior process runs along the dorso-lateral side of the body and forms the primary tentacular neurite bundle. At this stage, the paired ventral neurite bundle contains six bipolar perikarya, which contact each other via thin longitudinal neurites. These bipolar perikarya form the longitudinal neurite bundles which pass from the oral ring to the tentacular ridge (Figure 9H). Second, two groups of latero-ventral perikarya, each consisting of two or three perikarya (the lower ventro-lateral perikarya), are located near the tentacular ridge (Figure 9E). Third, two large perikarya arise on the ventro-lateral side near the site of contact between the preoral lobe and the hyposphere (the upper ventro-lateral perikarya) (Figure 9F, J). These perikarya connect to the marginal neurite bundle via the thin precursors of the ventro-lateral neurites (which is a part of the marginal neurite bundle and which continues into the oral field) (Figure 9J). Perikarya in the midgut are located near the pyloric sphincter; they are bipolar and triangular with a wide basal part that forms two processes that extend around the midgut (Figure 9K). Radial neurites of the preoral lobe are concentrated along its midline and run along the radial muscles (Figure 10D). At this stage, two to five radial neurites with median perikarya can be detected in the preoral lobe.

**Figure 11:**
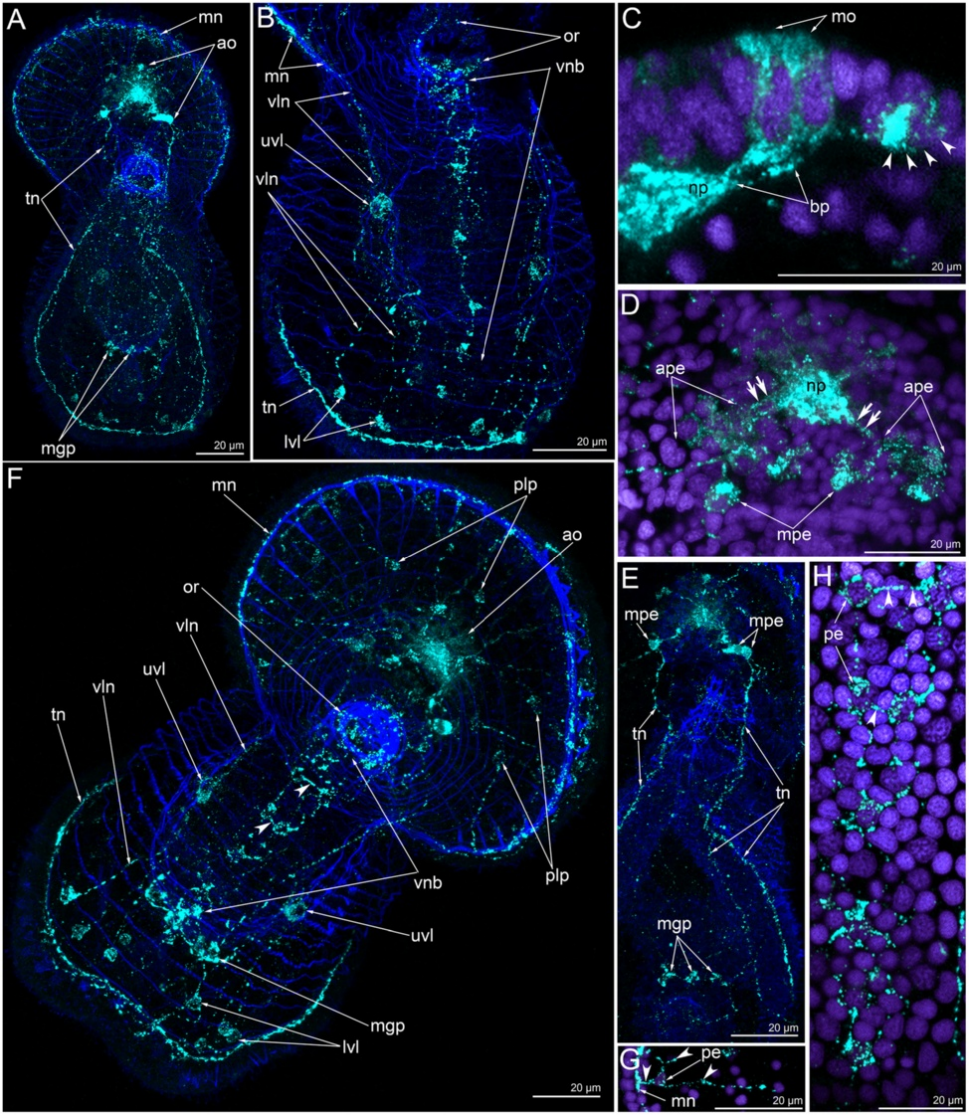
**Organization of the FMRFamide-like immunoreactive nervous system in 5-day-old (A-E) and 6-day-old (F-H) larvae.** Z-projections of larvae after double and triple staining with antibodies against FMRFamide (cyan), as well as staining with phalloidin (blue), and Hoechst (violet). Apical is to the top in all cases except for E, where it is to the upper right. (**A**) Overview showing apical organ (ao), marginal neurite bundle (mn), tentacular neurite bundle (tn), and midgut perikarya (mgp); dorsal view. (**B**) Oral field and basal part of the preoral lobe of a larva with oral nerve ring (or), marginal neurite bundle (mn), ventral neurite bundles (vnb), upper ventro-lateral perikarya (uvl), ventro-lateral neurites (vln), and lower ventro-lateral perikarya (lvl). Ventral view; the image is composed of the most ventral optical sections. (**C**) Lateral part of the apical organ with monopolar perikarya (mo), their basal processes (bp), neuropil (np), and bipolar or multipolar perikaryon (arrowheads). (**D**) Dorsal part of the apical organ with main bipolar or multipolar perikarya (mpe) and two groups of additional bipolar or multipolar perikarya (ape) that connect to the neuropil (np) via a prominent neurite bundle (double arrows). (**E**) Dorsal side of a larva with thin tentacular neurite bundle (tn) that arises from main bipolar or multipolar perikarya (mpe) and then forms two branches. Midgut perikarya (mgp) are shown. The micrograph contains the most dorsal optical sections only. (**F**) Overview of the musculature and FMRFamide-like immunoreactive nervous system showing apical organ (ao), perikarya of the preoral lobe (plp), marginal neurite bundle (mn), oral ring (or), ventral neurite bundles (vnb) with commissures (arrowheads), ventro-lateral neurites (vln), upper ventro-lateral perikarya (uvl), lower ventro-lateral perikarya (lvl), tentacular neurite bundle (tn), and midgut perikarya (mgp). Ventral view. (**G**) Part of the preoral lobe with marginal neurite bundle (mn) and perikaryon (pe) with neurites (arrowheads). (**H**) Ventral neurite bundleswith paired perikarya (pe) and commissures (arrowheads). Ventral view; four most ventral optical sections were used only to produce this image.

Five-day-old larvae contain all the neural elements observed in younger larvae (Figure 11F). The ventral neurite bundles, two large upper ventro-lateral perikarya, and two groups of lower ventro-lateral perikarya innervate the epidermis of the oral field (Figure 11B). The apical organ becomes more complex. The number of monopolar perikarya increases, but their shape does not change (Figure 11C). The number of bipolar perikarya also increases (Figure 11D). In addition to the two main (first) perikarya, two lateral groups of bipolar perikarya develop. These perikarya stain weakly; they form two lateral groups which connect to the neuropil via a prominent neurite bundle (Figure 11D). The main perikarya stain brightly and give rise to the tentacular neurite bundle. The entire tentacular neurite bundle appears in the 5-day-old actinotroch (Figure 11A). In some larvae, the tentacular neurite bundle bifurcates into two branches, one below and one above the tentacular ridge (Figure 11E).

In 6-day-old larvae, new perikarya appear adjacent to the paired ventral neurite bundle and in the epidermis of the preoral lobe (Figures 5E, 10E, 11F). In the latter case, most of the perikarya form two main neurites: one passes into the neuropil and the other connects to the marginal neurite bundle (Figure 11G). In some cases, the perikarya of the preoral lobe form more than two neurites, which spread into the preoral lobe epithelium (Figure 11G). The perikarya of the ventral neurite bundles form two longitudinal rows, which contact each other via thin commissures (Figure 11F, H). There are at least two commissures between the paired perikarya of the two longitudinal rows (Figures 10G, 11F, H). A new and large perikaryon appears near the mouth, in the epidermis of the oral field (Figure 10F). At this stage, the upper ventro-lateral perikarya form thin longitudinal ventro-lateral neurites, which contact the lower ventro-lateral perikarya (Figures 10F, 11F).

**Figure 12:**
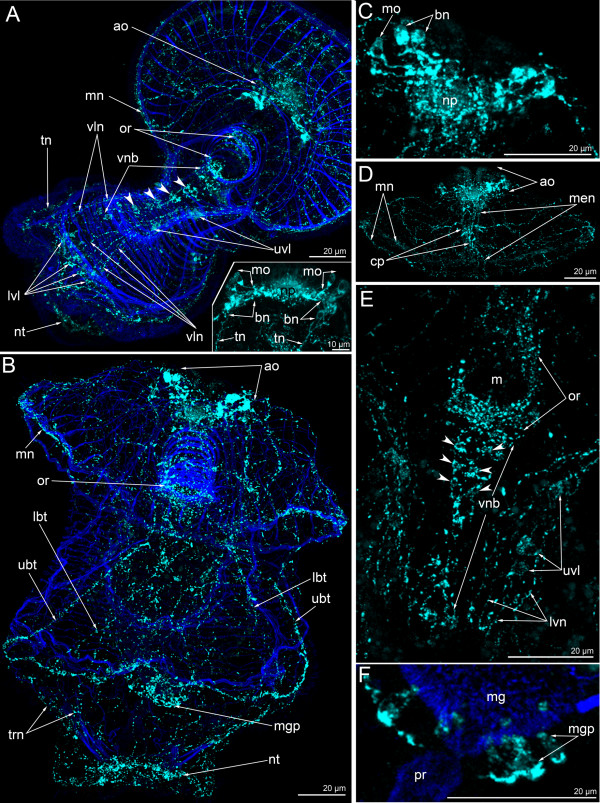
**Organization of the FMRFamide-like immunoreactive nervous system in 13-day-old (A, A-insert) and 24-day-old (B-F) larvae, respectively.** Z-projections of larvae after mono- and double staining with antibodies against FMRFamide (cyan), as well as staining with phalloidin (blue). Apical is to the top in all cases except for A, where it is to the upper right. (**A**) Overview of the musculature and FMRFamide-like immunoreactive nervous system showing apical organ (ao), marginal neurite bundle (mn), oral ring (or), ventral neurite bundles (paired perikarya are shown by arrowheads), upper ventro-lateral perikarya (uvl), ventro-lateral neurites (vln), lower ventro-lateral perikarya (lvl), tentacular neurite bundle (tn), and neurites of the telotroch (nt). Ventral view. (A-insert) Apical organ with two groups of monopolar perikarya (mo) and bipolar or multipolar perikarya (bn) that give rise to the tentacular neurite bundle (tn). Dorsal view. (**B**) Overview showing apical organ (ao), marginal neurite bundle (mn), oral ring (or), upper branch of the tentacular neurite bundle (ubt), lower branch of the tentacular neurite bundle (lbt), midgut perikarya (mgp), and neurites of the telotroch (nt). Dorsal view. (**C**). Ventral view of the apical organ with neuropil (np), monopolar perikarya (mo), and bipolar or multipolar perikarya (bn). (**D**) Ventral view of the preoral lobe showing apical organ (ao), marginal neurite bundles (mn), and median neurite bundle (men) with concentration of perikarya (cp). (**E**). Ventral view of the oral field with prominent oral nerve ring (or), ventral neurite bundles (vnc; paired perikarya are shown by arrowheads), upper ventro-lateral perikarya (uvl), and ventro-lateral neurites (vln). (**F**) Part of the proctodaeum (pr) and midgut (mg) with perikarya (mgp); dorsal view.

In 13-day-old larvae, the number of upper ventro-lateral perikarya increases to three or four (Figure 12A). Accordingly, the number of thin longitudinal neurites and the number of lower ventro-lateral perikarya also increases. At this stage, the connection is evident between the marginal neurite bundles of the preoral lobe, the upper ventro-lateral perikarya, the ventro-lateral longitudinal neurites, and the lower ventro-lateral perikarya (Figure 12A). 13-day-old larvae have a well-developed nerve net around the mouth that is connected to the paired ventral neurite bundles on its lateral side (Figure 12A). The ventral neurite bundles comprise seven or eight paired perikarya. The paired perikarya lie very close to each other, and the commissures cannot be recognized (Figure 12A). Larvae at this stage have primordial tentacles, and the tentacular neurite bundle forms loops into each primordium. A 13-day-old larva has a short trunk and the telotroch appears around the anus. Monopolar and bipolar or multipolar perikarya of the apical organ form two lateral clusters (Figure 12A-insert). Monopolar perikarya occupy the ventro-lateral epidermis of the apical plate, on the both sides of the neuropil. Bipolar or multipolar perikarya are located in the dorso-lateral epidermis of the preoral lobe. These perikarya form a compact group with three or five perikarya, which produce dorsal neurites — the tentacular neurite bundle (Figure 12A-insert).

In 24-day-old larvae, the FMRFamide-like immunoreactive nervous system is highly complex and contains numerous thin neurites (Figures 5F, 10H, I, 12B). The most prominent nervous element is the apical organ. It contains monopolar and bipolar or multipolar perikarya (Figure 12C). Monopolar perikarya are triangular with a narrow apical part and a wide basal part; the latter produces one basal process that extends into the neuropil. As before, the bipolar or multipolar perikarya form two dorso-lateral groups that contain between three and seven perikarya (Figure 12C). The dorso-lateral parts of the tentacular neurite bundle arise from the dorsal groups of the bipolar or multipolar perikarya of the apical organ. The tentacular neurite bundle passes along the tentacular ridge (Figure 10I). On the dorsal side, the tentacular neurite bundle contains upper and lower branches (Figure 10I). On the median line of the preoral lobe, the median neurite bundle appears (Figure 10J). It contains several neurites and perikarya. The latter are concentrated in the central part of the median neurite bundle (Figure 12D). Radial and circular neurites and two marginal neurite bundles innervate the preoral lobe. Both marginal neurite bundles split into numerous thin neurites where the preoral lobe merges with the hyposphere. The anterior marginal neurite bundle continues into ventro-lateral neurites of the oral field, whereas the posterior marginal neurite bundle passes into the lateral neurites of the oral field (Figure 10I). Thus, there are two pairs of groups of neurites that pass from the preoral lobe to the tentacular neurite bundle and that innervate the lateral and ventro-lateral sides of the oral field. The ventral side of the oral field is innervated by the paired ventral neurite bundle, which contains 15–17 bipolar perikarya (Figure 12E). Some of these perikarya are paired, but commissures are not present. The ventral neurite bundles connect to the oral ring. The latter is very complex and contains numerous neurites and perikarya. The neurites spread along the esophagus and form the cylindrical nerve net with a long dorsal side and a short ventral side (Figure 10H). There are six large perikarya on the dorsal side of the esophagus, whereby three are situated on the dorsal wall of the cardiac sphincter (Figure 10K). At this stage, there are two large perikarya, which are located in the epidermis of the oral field near the mouth (Figure 10I, K). These FMRFamide-reactive perikarya form two or three processes, some of which are in contact with the oral nerve ring (Figure 10K; see also [23]). The number of perikarya in the midgut increases to as many as 8–10 (Figure 12F). They form a circle around the midgut near the pyloric sphincter. The telotroch is innervated by the nerve ring, which passes along its external perimeter (Figure 10I). The trunk and dorsal body region are innervated by thin neurites (Figure 10I).

## Discussion

### The structure of the larval apical organ in lophotrochozoans and deuterostomes

The phoronid larval nervous system was first described on the basis of immunocytochemistry by Hay-Schmidt [16], who analyzed individual stages of early embryos and young larvae of *Phoronis vancouverensis*. According to these and our data, the first perikarya appear in the apical plate. The differentiation of the first perikarya in the apical organ is a common feature for numerous – but not all – lophotrochozoans ([24-27]; but see [28-30]). As is the case in other lophotrochozoans, the initial serotonin-like immunoreactive perikarya in the apical organ are flask-shaped in phoronid embryos. They possess a thin basal process and are retained during the entire larval development. The organization of the apical organ becomes more complex with age, as is indicated by the dramatic increase in the number of cells that constitute the apical organ.

In *Phoronopsis harmeri* larvae, four types of perikarya form the apical organ: monopolar and bipolar (or multipolar) serotonin-like immunoreactive as well as monopolar and bipolar (or multipolar) FMRFamide-like immunoreactive. Within the apical organ, only the monopolar serotonin-like immunoreactive perikarya are flask-shaped. In young larva, there are 20–25 monopolar serotonin-like immunoreactive perikarya, which form a horseshoe-shaped (U-shaped) field with two dorsal branches. These cells are monociliated, flask-shaped, and their basal processes pass to the centre of the apical organ and form the neuropil. Bipolar (or multipolar) serotonin-like immunoreactive perikarya and some of their processes form the posterior-most layer of the apical organ. Their perikarya form two (left and right) groups with 5–7 perikarya, which are located directly under the monopolar perikarya. In 24-day-old larva, the neuropil, which is composed of the neurites of the bipolar (or multipolar) serotonin-like immunoreactive perikarya, is subdivided into two portions (left and right). The monopolar FMRFamide-like immunoreactive perikarya form one group each on the left and right side of the apical organ, respectively. Bipolar or multipolar FMRFamide-like immunoreactive perikarya are strongly stained and form two dorso-lateral groups, which occupy the most dorsal area of the apical organ. Groups of bipolar or multipolar FMRFamide-like immunoreactive perikarya connect to the tentacular FMRFamide-like immunoreactive neurite bundle and appear earlier than monopolar FMRFamide-like immunoreactive perikarya.

Hay-Schmidt [17] found three types of perikarya in the apical organ of *Phoronis muelleri*. These are monopolar and bipolar or multipolar serotonin-like immunoreactive perikarya as well as monopolar FMRFamide-like immunoreactive perikarya. In early larvae of *P. vancouverensis*, the apical organ consists of two types of perikarya: monopolar and bipolar or multipolar serotonin-like immunoreactive cells [16]. Interestingly, the apical organ neither contains monopolar nor bipolar (multipolar) FMRFamide-like immunoreactive cells. In *Phoronis pallida*, two types of serotonin-like immunoreactive perikarya were found in the apical organ: bipolar ciliate and nonciliate [19].

Taken together, the data available show that the apical organs of phoronid larvae have a much more complex architecture than that of other lophotrochozoans. It consists of at least four cell types and contains approximately 30–50 serotonin-like immunoreactive and 16–20 FMRFamide-like immunoreactive perikarya. The majority of the serotonin-like immunoreactive cells consist of monopolar or (presumably) bipolar flask-shaped perikarya, which form a U-shaped mass.

In most lophophotrochozoan larvae, the apical organ consists of only a few serotonin-like immunoreactive flask-shaped cells (annelids: [28-31]; molluscs: [32-34]; polyclad flatworms: [35]; ectoprocts: [36,37]; brachiopods: [9,38]; see [27] for review). Such a simple apical organ can therefore be regarded as a basal feature of spiralian protostomes [24,27,39]. However, there are deviations from this general pattern. The high degree of complexity of the apical organ of entoproct and polyplacophoran larvae has been considered one of several apomorphies of monophyletic Entoprocta-Mollusca (i.e., Tetraneuralia; [27]). On the other hand, some polychaete annelids or ectoprocts seem to lack flask-shaped cells in the apical organ altogether [40,41].

The organization of the apical organ of lecithotrophic brachiopod larvae differs from that of the swimming planktotrophic juveniles of glottiid brachiopods. While the latter have numerous, probably non-flask-shaped cells, the former have four or more (eight?) serotonin-like immunoreactive flask-shaped cells [9,38,42], again suggesting that these cells belong to the groundpattern of Lophotrochozoa.

In the deuterostome larvae investigated so far (enteropneust tornariae and echinoderm larvae), the apical organ consists of more than ten or fifteen serotonin-like immunoreactive perikarya, the shape of which probably being typically flask-shaped [43-45].

Accordingly, two scenarios concerning the evolution of complexity in invertebrate apical organs appear principally possible. Either, the last common ancestor of protostomes and deuterostomes had a simple apical organ and its complexity evolved three times independently: once in the entoproct-molluscan (tetraneuralian) lineage, once in phoronids, and once in the deuterostomes, or a complex apical organ was present in the last common ancestor and has been retained in the three above-mentioned clades, while it was subsequently reduced multiple times independently in the remaining spiralians.

### Comparison of the overall neural architecture of phoronid larvae with other lophotrochozoan and deuterostomian larvae

In young *P. harmeri* larvae, the serotonin-like immunoreactive nervous system consists of four perikarya-containing subsets: the apical organ, a group of six to eight perikarya distributed along the edge of the preoral lobe, frontal organ, and a group containing the oral nerve ring with ventro-lateral perikarya, which connect to the ventral neurite bundles. Perikarya of the preoral lobe interconnect with the serotonin-like immunoreactive marginal neurite bundles of the preoral lobe and can not be recognized in late stages. However, in *P. muelleri* larvae, these perikarya are retained in late stages and are concentrated along the midline of the edge of the preoral lobe (note that the serotonin-like immunoreactive marginal neurite bundle is absent in *P. muelleri* larvae; [17]).

In older *P. harmeri* larva, a fourth serotonin-like immunoreactive perikaryon appears in the frontal organ. It contains several perikarya which connect to the serotonin-like immunoreactive median neurite bundle of the preoral lobe. The frontal organ has been described for other phoronid larvae as well [46] and contains serotonin-like immunoreactive perikarya [21]. The median neurite bundle of the preoral lobe is found in all phoronid larvae studied so far, except for young stages of *P. vancouverensis*[16]. In the larva of *P. harmeri*, this neurite bundle emerges from the left group of serotonin-like immunoreactive bipolar (or multipolar) perikarya.

*P. harmeri* is the only phoronid larva to date for which a paired ventral neurite bundle has been described ([22], herein). It consists of two longitudinal neurite bundles, each being associated with several bipolar (or multipolar), paired perikarya. The ventral neurite bundles are interconnected by serially repeated commissures. Such a ventral nervous system is common for many larval (and/or) adult spiralians, and although the number of the ventral neurite bundles may vary between and even within various phyla, a paired ventral neurite bundle with serially arranged commissures is usually considered basal for lophotrochozoans [27]. Accordingly, the larva of *P. harmeri* appears to have retained this neural structure, while it was lost in the adults as well as in adults and larvae of the other phoronid species investigated so far. At the same time, the paired ventral neural bundle of *P. harmeri* larvae differs from the one of typical spiralian larvae (for details see [22]).

In all phoronid larvae studied to date, the serotonin-like immunoreactive tentacular neurite bundle is a prominent structure. In *P. harmeri* larvae, two dorso-lateral branches of the tentacular neurite bundle are connected each other via a dorsal commissure, which seems to be lacking in other phoronid species. According to some data [21], there are two tentacular neurite bundles: one minor (extends along tentacles over them) and one main bundle (extends along tentacles under them).While in phoronid larvae the tentacular neurite bundle forms contact with the apical organ, the apical organ never directly connects to the neurite bundles that innervate the ciliated bands in spiralian trochophores [25,29,30]. In ambulacrarian larvae (chinoderms and hemichordates), however, the apical organ is also connected to the neurite bundles of ciliated bands, as is the case in the phoronids [45-48]. Moreover, according to some data [47,48], the apical organ of echinoderms arises as a bilaterally symmetrical nerve plexus, which is generated by neurite bundles of ciliated bands. In ambulacraria, the apical organ arises after or simultaneously with the neurite bundles and perikarya associated with the ciliary bands [44,47], while in actinotroch larvae, serotonin-like immunoreactive neurites innervating the ciliated bands are formed after the establishment of the apical organ. Accordingly, the left and right branches of the apical organ give rise to the two lateral tentacular neurite bundles of the phoronid larva. The telotroch in phoronid larvae is innervated by two (in *P. harmeri*) or one (in *P. muelleri*) serotonin-like immunoreactive circular neurite bundles.

Thus, in all actinotrochs studied, all ciliated bands are innervated by prominent serotonin-like immunoreactive neurite bundles, some of them (e.g., the marginal neurite bundle) containing serotonin-like immunoreactive perikarya. Scattered perikarya associated with neurites that innervate ciliated bands are known from larvae of enteropneust and echinoderm deuterostomes [44,45,49,50].

The larval FMRFamide-like immunoreactive nervous system is very complex in *P. harmeri*. In this species, the FMRFamide-like immunoreactive nervous system appears to be associated with the major muscle systems. The first neurites appear in the epidermis of the apical plate and then along the edge of the preoral lobe (marginal neurite bundle) in young actinotrochs and is retained in late stages. In older actinotrochs, there are two FMRFamide-like immunoreactive marginal neurite bundles [16,17], herein. These bundles extend into the oral field and furcate into individual neurites, which ventrolaterally connect to two groups of perikarya. In most previous reports [16,19,21], the FMRFamide-like immunoreactive system of actinotrochs does not seem to contain any perikarya even in the apical organ. However, in *P. harmeri*, there are five main groups of perikarya, which are located in the oral field (three groups), the preoral lobe, and the midgut. Perikarya in the oral field were found in *P. muelleri* larvae. They are multiplied and form the layer along the upper border of the tentacular ring [17]. In larvae of *P. harmeri* and *P. muelleri*, there are perikarya along the medio-ventral side of the oral field. In young larvae of *P. harmeri*, these perikarya are part of the paired ventral neurite bundle. In older larvae, the commissures interconnecting the ventral neurite bundles were not found [22]. Another group of perikarya is formed by perikarya which are scattered in the epidermis of the preoral lobe [17], herein. Neurites of these perikarya are associated with the radial muscles of the preoral lobe. Perikarya in the midgut were described for the first time in *P. harmeri*[23]. The FMRFamide-like immunoreactive tentacular neurite bundle was found in all actinotrochs studied to date [16,17,19,21]. It runs under the tentacles and has two pairs of dorsal branches in *P. harmeri*. The FMRFamide-like immunoreactive nerve ring around the telotroch is described for the first time herein.

The digestive tract of the actinotroch larva is associated with serotonin-like immunoreactive and FMRFamide-like immunoreactive perikarya and neurites (for details see [23]). The most prominent nervous elements were found in the esophagus (oral nerve ring), midgut (FMRFamide-reactive perikarya and circular neurites), and the proctodaeum of actinotroch larvae. Some bilaterian planktotrophic larvae have a nerve ring around the mouth (esophagus), but the innervation of other parts of the digestive tract is still unclear. In trochozoan planktotrophic larvae, there is an oral nerve ring, which connects to apical organ via esophageal connectives [25,28-30]. Some deuterostome larvae have serotonin-like immunoreactive, FMRFamide-like immunoreactive, and SALFamidergic perikarya and neurites in the esophagus, stomach, and pylorus [43,45]. These prominent neural features are directly correlated with the long planktotrophic life in the water column of these organisms.

## Conclusion

*P. harmeri* has the most complex larval serotin- and FMRFamide-like immunoreactive nervous systems of all phoronids studied to date. The gross anatomy of the nervous system of the actinotroch larva combines characteristics of both lophotrochozoan and deuterostome larvae. On the one hand, the phoronid apical organ consists of flask-shaped serotonin-like immunoreactive monopolar perykaria, which most likely constitute a lophotrochozoan apomorphy [9]. On the other hand, the complexity of the phoronid apical organ, which contains more than 40 serotonin-like immunoreactive perikaria, resembles that of some deuterostome larvae. The distant relationship of phoronids and deuterostomes suggests independent origin of these complex apical organs, although a complex apical organ at the base of the protostome-deuterostome split with subsequent independent simplification in most spiralians cannot be ruled out. The finding of a paired ventral neurite bundle with serially arranged commissures suggests that such a neural feature was part of the ancestral phoronid – and most likely also the ancestral lophotrochozoan – bodyplan, which was secondarily lost in adult phoronids, probably in connection with the acquired sedentary lifestyle.

## Methods

### Animals

Adult *Phoronopsis harmeri* were collected from May to June 2010 in Coos Bay, Oregon, USA, from intertidal sandy sediments. Fertilized eggs, which were extracted from reproductive females by opening the trunk, were kept in glass beakers containing filtered sea water; the temperature of the egg suspension was maintained at 13-14°С by keeping the beaker partially submerged in running sea water on a laboratory bench. Under these conditions, embryos developed normally. Within 15 min of exposure to sea water, two polar bodies were formed and cleavage commenced. Stages of development were monitored with a stereo microscope. The apical tuft and large apical plate became visible 16 h after formation of the polar bodies. This stage was determined as the initiation of neurogenesis. Larval cultures had a density of two larvae per 3–4 ml filtered sea water. Larvae were fed mixtures of *Rhodomonas lens* and *Chaetoceros calcitrans* and 75% of the sea water was changed every 2 days. Consecutive stages from the 16-h-old coeloblastula to the 6-tentacled larvae (24 days old) were photographed from living specimens with a Leica DFC 400 camera mounted on an Olympus BX51 microscope equipped with DIC optics.

### Immunocytochemistry

At 4 h intervals (up to the early actinotrocha), specimens were prepared for immunocytochemistry and confocal laserscanning microscopy (CLSM). Embryos and larvae were narcotised in 0.34 M MgCl_2_ (Fisher Scientific, Pittsburgh, PA, USA), fixed for 60 min in a 4% paraformaldehyde (Electron Microscopy Science, Hatfield, PA, USA) solution in filtered sea water, and washed (three times x 15 min) in phosphate buffer (pH 7.4) (Fisher Scientific) with Triton X-100 (0.1%) (Fisher Scientific, Pittsburgh, PA, USA) and 0.1% albumine bovine (PBT/BSA) (Sigma-Aldrich, St. Louis, MO, USA). Nonspecific binding sites were blocked with 5% normal donkey serum (Jackson ImmunoResearch, Newmarket, Suffolk, UK) in PBT/BSA for 2 h at room temperature (RT). Subsequently, the specimens were washed in PBT/BSA, the larvae were transferred into primary antibody (either rabbit anti-serotonin or rabbit anti-FMRFamide, ImmunoStar, Hudson, WI, USA) solutions 1:800 in PBT/BSA and incubated overnight at 4 °C. Specimens were washed (three times x 15 min) in PBT/BSA and then exposed to the secondary antibody, donkey anti-rabbit-Alexa Fluor 488 (Invitrogen, Grand Island, NY, USA), at a dilution of 1:1000 for 2 h at RT in the dark. Then, the specimens were washed in PBT/BSA and incubated in a mixture of rhodamine-conjugated phalloidin (1:50) (Fisher Scientific, Pittsburgh, PA, USA) and Hoechst (1:1000) (Fisher Scientific, Pittsburgh, PA, USA) for 1 h at RT in the dark. In the following, they were washed in PBS (three times x 15 min), mounted on a cover glass covered with poly-L-lysine (Sigma-Aldrich, St. Louis, MO, USA), and embedded in Murray Clear or Vectashield (Vector Laboratories Inc., Burlingame, CA, USA). Specimens were viewed with an Olympus confocal microscope (OIMB, OR, USA). Z-projections were generated using the programme Image J version 1.43. Three-dimensional reconstructions were generated using Amira version 5.2.2 software (Bitplane, Zurich, Switzerland).

Since the larval intestine shows strong background signal, it may produce fluorescence on different channels, and some of our micrographs contain nonspecific staining of the intestine. We therefore conducted two experiments to discern autofluorescence of the intestine from true immunocytochemical signal. We treated the larvae according to the protocol described above but omitted either the primary or the secondary antibodies. In both cases, we detected autofluorescence of the intestine on 488, 594 and 633 channels; the samples were devoid of any other signal, however (data not shown).

## Competing interests

The authors declare that they have no competing interests.

## Authors’ contributions

ET designed and coordinated research, performed research, analyzed data, and prepared all figures. AW contributed to interpretation and discussion of the data. ET and AW wrote the manuscript. All authors conceived the study, read, and approved the final version of the manuscript.
